# Targeted genomic analysis reveals widespread autoimmune disease association with regulatory variants in the TNF superfamily cytokine signalling network

**DOI:** 10.1186/s13073-016-0329-5

**Published:** 2016-07-19

**Authors:** Arianne C. Richard, James E. Peters, James C. Lee, Golnaz Vahedi, Alejandro A. Schäffer, Richard M. Siegel, Paul A. Lyons, Kenneth G. C. Smith

**Affiliations:** Department of Medicine and Cambridge Institute for Medical Research, The University of Cambridge, Box 139, Cambridge Biomedical Campus, Hills Road, Cambridge, CB2 0XY UK; Autoimmunity Branch, National Institute for Arthritis and Musculoskeletal and Skin Diseases, National Institutes of Health, Bethesda, MD 20892 USA; Department of Genetics, Institute for Immunology, Perelman School of Medicine, University of Pennsylvania, Philadelphia, PA 19104 USA; Computational Biology Branch, National Center for Biotechnology Information, National Institutes of Health, Bethesda, MD 20894 USA

**Keywords:** TNF superfamily, Autoimmunity, Autoinflammation, Genomics, eQTL, Gene set analysis, GWAS, Genetics

## Abstract

**Background:**

Tumour necrosis factor (TNF) superfamily cytokines and their receptors regulate diverse immune system functions through a common set of signalling pathways. Genetic variants in and expression of individual TNF superfamily cytokines, receptors and signalling proteins have been associated with autoimmune and inflammatory diseases, but their interconnected biology has been largely unexplored.

**Methods:**

We took a hypothesis-driven approach using available genome-wide datasets to identify genetic variants regulating gene expression in the TNF superfamily cytokine signalling network and the association of these variants with autoimmune and autoinflammatory disease. Using paired gene expression and genetic data, we identified genetic variants associated with gene expression, expression quantitative trait loci (eQTLs), in four peripheral blood cell subsets. We then examined whether eQTLs were dependent on gene expression level or the presence of active enhancer chromatin marks. Using these eQTLs as genetic markers of the TNF superfamily signalling network, we performed targeted gene set association analysis in eight autoimmune and autoinflammatory disease genome-wide association studies.

**Results:**

Comparison of TNF superfamily network gene expression and regulatory variants across four leucocyte subsets revealed patterns that differed between cell types. eQTLs for genes in this network were not dependent on absolute gene expression levels and were not enriched for chromatin marks of active enhancers. By examining autoimmune disease risk variants among our eQTLs, we found that risk alleles can be associated with either increased or decreased expression of co-stimulatory TNF superfamily cytokines, receptors or downstream signalling molecules. Gene set disease association analysis revealed that eQTLs for genes in the TNF superfamily pathway were associated with six of the eight autoimmune and autoinflammatory diseases examined, demonstrating associations beyond single genome-wide significant hits.

**Conclusions:**

This systematic analysis of the influence of regulatory genetic variants in the TNF superfamily network reveals widespread and diverse roles for these cytokines in susceptibility to a number of immune-mediated diseases.

**Electronic supplementary material:**

The online version of this article (doi:10.1186/s13073-016-0329-5) contains supplementary material, which is available to authorized users.

## Background

The tumour necrosis factor (TNF) cytokine and receptor superfamilies are composed of 18 ligands (TNFSF) and 29 receptors (TNFRSF), respectively, that share both structural and signalling characteristics [1–3]. Members of these superfamilies modulate immunological responses via co-stimulation, maturation and cell death signalling pathways. In addition, they play important roles in bone homeostasis, eccrine gland development and the nervous system. The expression of TNFSF and TNFRSF molecules is often limited to particular cell types and modulated by the maturation or activation status of these cells [4]. TNFRSF ligation has three broad consequences: cellular activation via TRAF family proteins leading to NF-κB and MAP kinase activity; caspase-dependent death via FADD and caspase-8; and caspase-independent necroptosis mediated by RIP1 and RIP3 kinases. Certain TNFRSF members recruit the adaptor protein TRADD, which subsequently signals through either TRAFs or FADD, depending on cellular context. In addition, decoy receptors within the superfamily can inhibit TNFSF signalling by binding specific ligands without initiating downstream signalling. Recent research has begun to shed light on additional complexities of these signalling pathways. For example, the molecules cFLIP, RIP3 and caspase-8 co-ordinately drive TNF signalling through TRADD to initiate survival, apoptotic or necroptotic pathways depending on their relative concentrations and/or activities [5].

Genetic studies have implicated TNFSF and TNFRSF members in immune-mediated diseases. Mendelian syndromes such as autoimmune lymphoproliferative syndrome (ALPS) and TNF receptor associated periodic syndrome (TRAPS) are caused by mutations in *FAS* (or other members of the FAS signalling pathway) and *TNFRSF1A*, respectively [6–8]. The mechanisms by which missense mutations drive these two syndromes differ: heterozygous dominant negative *FAS* mutations lead to defective signalling in ALPS patients [6], while heterozygous *TNFRSF1A* mutations in TRAPS patients result in endoplasmic reticulum retention of mutant proteins and exacerbated inflammatory signalling [9]. Mutations in TNFRSF members can also lead to common variable immunodeficiency (CVID): approximately 9 % of patients carry one or two variant alleles of *TNFRSF13B* (encoding TACI) [10] and a few patients carry biallelic mutations of *TNFRSF13C* (encoding BAFF-R) [11]. Although CVID is by definition an immunodeficiency, many CVID patients suffer from autoimmune diseases [12]. For example, heterozygous carriers of *TNFRSF13B* mutations are susceptible to autoimmunity via the failure of central tolerance to select against autoreactive B cells [13]. Genome-wide association studies (GWASs) of common autoimmune and autoinflammatory diseases have identified associations with single nucleotide polymorphisms (SNPs) near a quarter of the 88 autosomal genes encoding TNFSF cytokines, their receptors and downstream signalling molecules [14] (Additional files 1, 2 and 3). Many genetic variants in the TNFSF network are associated with multiple diseases and many diseases are associated with multiple variants in TNFSF network genes. Whether the same genetic variant truly underlies different diseases is likely to remain ambiguous until the causal variants are fine-mapped [15–17].

Increased expression of TNFSF and TNFRSF members has been observed in the serum and/or at the site of inflammation in patients with immune-mediated disease, including rheumatoid arthritis (RA) [18–20], inflammatory bowel disease (IBD) [21–25] and systemic lupus erythematosus (SLE) [26–28]. In addition, mouse models of both autoimmune disease and allergic asthma can be ameliorated by genetic or therapeutic blockade of numerous TNFRSF signalling pathways [29]. TNFSF pathogenicity in these diseases is further corroborated by the success of therapeutically targeting TNF [30] and TNFSF13B (BAFF) [31], as well as on-going development of therapeutics against additional family members [32]. Given that the majority of disease-associated genetic variants in TNFSF-related genes are non-coding and that expression of many of these genes is dysregulated in the same diseases, the question arises as to whether genetic variants directly drive pathogenic expression changes. Recent genome-wide expression quantitative trait loci (eQTL) studies have uncovered disease-associated SNPs that may regulate expression of nearby TNFSF and TNFRSF members in several primary leucocyte subsets [33–40]. In-depth studies of specific polymorphisms have revealed direct consequences on gene expression and occasionally downstream phenotype for disease-associated variants located near *TNFSF4* [41, 42], *TNFRSF1A* [43], *TNFSF14* [44], *CD40* [45], *TNFRSF6B* [46] and *TNFSF15* [47–50]. However, most of these studies focus on a single leucocyte subset or whole blood measurements.

**Fig. 1:**
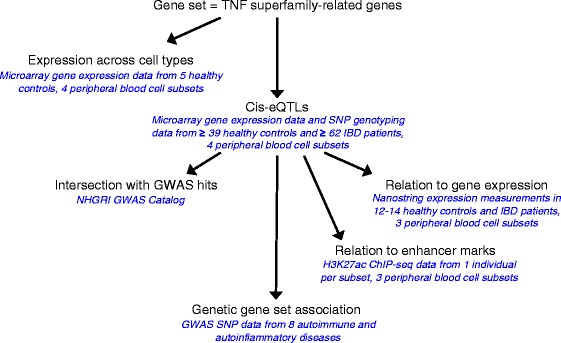
Flow chart of analyses. Flow chart demonstrates how results from each analysis feed into the next. Datasets analysed are listed in *blue italics*

Here we took a hypothesis-driven approach to investigate how genetic variants that regulate genes encoding TNFSF and TNFRSF members, as well as key downstream signalling molecules, influence disease susceptibility. Our workflow is depicted in Fig. 1. We examined regulation of these genes across peripheral blood leucocyte subsets by mapping eQTLs. Using these eQTL SNPs as genetic markers of TNFSF-related genes, we performed gene set association analysis with autoimmune and autoinflammatory diseases. This revealed widespread association with the TNFSF gene network.

## Methods

### GWAS Catalog search for TNFSF-related genes and intersection with eQTLs

Processing and analysis of data from the NHGRI GWAS Catalog [14] is described in Additional file 4: Supplemental Methods.

### Sorting peripheral blood subsets from individuals

Whole blood collection for this study was approved by the Cambridgeshire 3 Research Ethics Committee (08/H0306/21). Written informed consent was obtained from all participants. Whole blood from healthy controls and individuals with newly diagnosed, flaring Crohn’s disease (CD) or ulcerative colitis (UC) was separated into peripheral blood leucocyte subsets by magnetic bead-based positive selection [51, 52] as described in Additional file 4: Supplemental Methods. CD4^+^ T cells, CD8^+^ T cells, CD14^+^ monocytes and CD16^+^ neutrophils were used in this study, a subset of the samples described in [53].

### Gene expression measurements and data processing

RNA (200 ng) from each sample was prepared for Human Gene 1.1 ST 96-Array (Affymetrix) using the Ambion WT Expression Kit and GeneChip WT Terminal Labeling and Controls Kit (Affymetrix). These samples were run in batches of 96 on a Gene Titan Multi-Channel (MC) Instrument (Affymetrix). Gene expression data are available through ArrayExpress, accession numbers E-MTAB-3554 ([53] eQTL analysis) and E-MTAB-4887 (comparison across leucocyte subsets in healthy controls). Processing of gene expression data is described in Additional file 4: Supplemental Methods.

### Genotyping for eQTL analysis

DNA samples were extracted with the Qiagen All-Prep DNA/RNA Mini kit from peripheral blood cells. DNA was genotyped on the Illumina Beadchip HumanOmniExpress-12v1 platform at the Wellcome Trust Sanger Institute in two batches. These data have been deposited in the European Genome-phenome Archive (EGA; accession number EGAS00001001251 [53]) and are available on request. Genotype calls were made using GenoSNP software. Processing of genotype data is described in Additional file 4: Supplemental Methods.

### eQTL analysis

Details of samples used in eQTL analyses are tabulated in Additional file 5. *Cis*-eQTL mapping to autosomal TNFSF-related genes was carried out in each cell type separately using the All.cis function of the GGtools Bioconductor package [54]. This method fits a generalised linear model with expression as the dependent variable and then performs score tests (one degree of freedom asymptotic chi-squared tests) for the addition of genotype to the model. *P* values were calculated in a one-tailed test from the chi-squared scores. Probe set location annotation was based on Ensembl release 71 and SNPs were annotated with SNPlocs.Hsapiens.dbSNP.20120608 [55]. SNPs were filtered for minor allele frequency above 5 %. False discovery rate (FDR) was estimated by sample label permutation [56] with a threshold of 10 % applied for significance. Because our ultimate goal in this re-analysis study was to perform gene set disease association analysis, we wished to uncover additional eQTLs to tag our TNFSF-related genes. We felt that a 10 % FDR threshold provided a reasonable compromise between maximising eQTL discovery and minimising false positives. We found 320 permutations to be more than sufficient to achieve FDR stability (Additional file 4: Figure S1a). Regulatory elements have been shown to primarily reside within 50 kbp of the transcription start and end sites of a gene [57]. By varying the radius from 50 kbp to 1 Mbp while controlling the FDR at 10 %, we found that the number of eQTL discoveries increased from 50 to 100 kbp and then declined at larger radii tested (Additional file 4: Figure S1b). Thus, for each gene, SNPs within the region from 100 kbp upstream to 100 kbp downstream of the gene were designated as *cis*. For each *cis*-eQTL in the combined IBD and healthy control cohort, we also performed linear regression of expression on SNP genotype in patients and controls separately. We then plotted the coefficients of the genotype term for each eQTL in IBD patients versus healthy controls to compare effect sizes and directions. See Additional file 4: Supplemental Methods for variable selection at loci with multiple significant *cis*-eQTL SNPs.

### Nanostring nCounter measurements and data processing

RNA was previously measured by the nCounter Analysis System (Nanostring Technologies) [58]. See Additional file 4: Supplemental Methods for sample, measurement, and normalisation details.

### Intersection of H3K27ac chromatin immunoprecipitation sequencing data with eQTLs

H3K27ac chromatin immunoprecipitation sequencing (ChIP-seq) and ChIP input DNA sequencing .bed files for CD4^+^ T cells, CD8^+^ T cells and CD14^+^ monocytes were obtained from the NIH Roadmap Epigenomics Project datasets [59] available through the Gene Expression Omnibus (GEO) database [60]: H3K27ac CD4+ CD25− primary cells, GSM997239; input CD4+ CD25− primary cells, GSM1112781; H3K27ac CD8 primary cells, GSM1102781; input CD8 primary cells, GSM1102806; H3K27ac CD14 primary cells, GSM1102782; input CD14 primary cells, GSM1102807. The available H3K27ac immunoprecipitated and input DNA sequencing data from CD4+ CD25− T cells were not from the same sample. Processing and analysis of H3K27ac ChIP-seq data are described in Additional file 4: Supplemental Methods.

### Hypothesis-driven genetic gene set analyses in previous GWAS datasets

Genetic studies typically examine association of individual SNPs with disease. This approach fails to exploit functional relationships between SNPs affecting the same gene or biological pathway. To address this, we performed gene set association analysis using data from previous GWASs. Previous GWAS datasets are detailed in Additional file 6. Processing of these genetic data is described in Additional file 4: Supplemental Methods. SNPs to represent the TNFSF-related gene set were chosen as follows. In each cell subset, we selected SNPs in linkage disequilibrium (LD) r^2^ ≥ 0.8 with the strongest significant *cis*-eQTL SNP for each TNFSF-related gene. This was performed using 1000 Genomes Phase 1 EUR population vcf files [61] in PLINK [62, 63]. In each GWAS dataset, we then extracted all of these SNPs that were present on the SNP chip used. Next, we filtered the SNPs for relative independence (multiple correlation coefficient ≤ 0.33) to make our SNP set representative of TNFSF-related genes, referred to hereafter as the TNFSF eQTL SNP set. In each GWAS dataset, hypothesis-driven gene set association analysis was based on that of Sun et al. [64] as follows. We calculated chi-squared allelic case-control association statistics and inflation factor (λ) for the TNFSF eQTL SNP set. The same independence filtering and association testing was then performed genome-wide. Qq-plots were compared between TNFSF eQTL SNPs and SNPs genome-wide. λ_1000_ values were calculated by rescaling λ for 1000 cases and 1000 controls:

$$ {\lambda}_{1000}=1+\left(\lambda -1\right)\bullet \frac{\frac{1}{n_{cases}}+\frac{1}{n_{controls}}}{\frac{1}{1000}+\frac{1}{1000}} $$

To calculate a self-contained gene set association statistic [65, 66], phenotypes were permuted 10,000 times and chi-squared disease association statistics were calculated in each permuted dataset for the TNFSF eQTL SNP set. We then summed chi-squared scores across SNPs in the original data and in each permuted dataset. Empirical *p* values were calculated as the fraction of summed scores from permuted datasets that were greater than that from the original data. A similar procedure was followed to estimate gene-level disease association. For each gene, the sum of chi-squared statistics was compared with the sum of chi-squared statistics in the permuted data to obtain an empirical *p* value. Gene-level *p* values were then adjusted for the multiple genes tested using the Benjamini–Hochberg method. Comparison of gene length with disease association *p* values revealed negligible impact of gene length on association statistics (Additional file 4: Figure S2).

## Results

### TNF superfamily-related genes are differentially regulated among leucocyte subsets

**Fig. 2:**
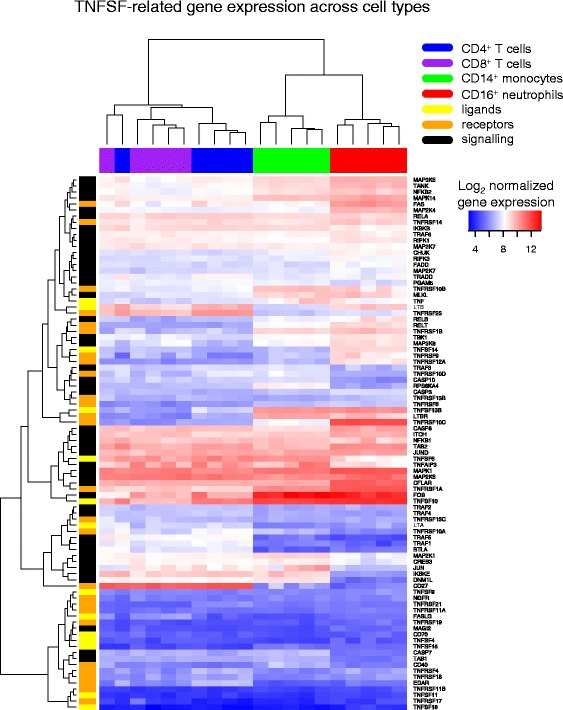
Expression of TNFSF-related genes differs across leukocyte subsets. Expression of TNFSF-related genes was measured across four cell subsets from five healthy controls by microarray. Expression values are hierarchically clustered. Cell types are coloured *blue* (CD4^+^ T cells), *purple* (CD8^+^ T cells), *green* (CD14^+^ monocytes) and *red* (CD16^+^ neutrophils). Genes are grouped by function and coloured *yellow* (TNFSF member ligands), *orange* (TNFRSF member receptors) and *black* (adaptors and signalling molecules in TNFSF signalling network)

We curated genes that encode members of the TNFSF and TNFRSF and their downstream signalling molecules from the literature (Additional file 2) and examined their expression in CD4^+^ T cells, CD8^+^ T cells, CD14^+^ monocytes and CD16^+^ neutrophils sorted from peripheral blood from five healthy individuals (Fig. 2). The signalling molecules downstream of superfamily receptors were generally expressed more broadly across cell subsets than TNFSF ligands or TNFRSF receptors. Hierarchical clustering of gene expression levels across cell types revealed cell type-specific expression, separating the lymphoid (CD4^+^ and CD8^+^ T cells) and myeloid (monocytes and neutrophils) lineages and clearly distinguishing monocytes from neutrophils. Such cell type clustering occurred even when only TNFSF, TNFRSF or signalling molecules were considered (Additional file 4: Figure S3).

**Fig. 3:**
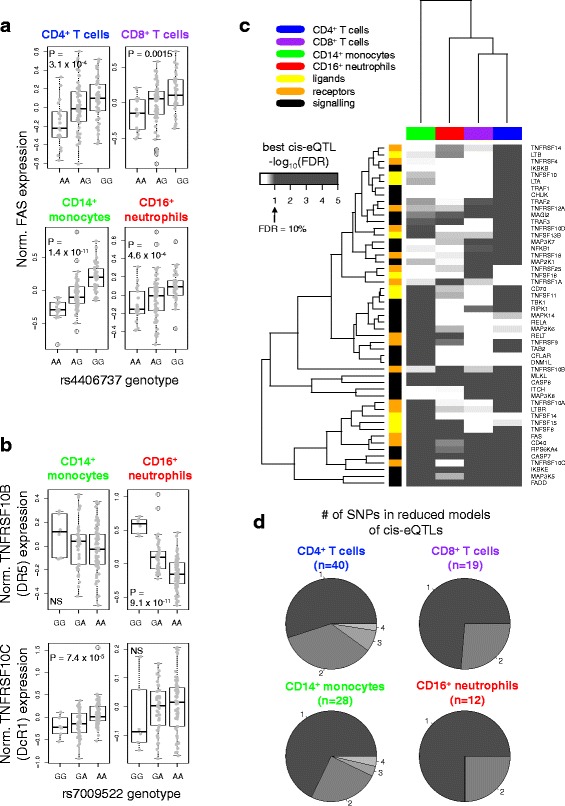
TNFSF-related genes are under extensive genetic regulation. **a** Normalised FAS expression in each cell subset is plotted against rs4406737 genotype. Association *p* values are indicated for eQTLs with FDR < 0.1. **b** Normalised TNFRSF10B and TNFRSF10C expression in monocytes and neutrophils is plotted against rs7009522 genotype. *NS* not significant. **c** TNFSF-related genes with a significant *cis*-eQTL (FDR < 0.1) in any cell type were extracted. For each gene, the SNP most significantly associated with expression in each cell type was extracted (best *cis*-eQTL) and the FDR corresponding to its *p* value in that dataset calculated. The plot depicts hierarchical clustering of the negative logarithm of these FDRs. *Colours* are as in Fig. 2. **d** The number of SNPs in *cis*-eQTL models after variable selection. Numbers around the *periphery* and *grey shades* indicate the number of eQTL SNPs remaining as predictors in the model for each gene. “n” indicates the number of genes with *cis*-eQTLs represented by each pie chart

To examine the relationship between genetic variation and expression of these TNFSF-related genes, we performed targeted *cis*-eQTL mapping in a previously analysed cohort of combined healthy controls and individuals with newly diagnosed IBD [53]. Although eQTLs have been mapped for TNFSF-related genes in genome-wide studies, our targeted approach reduced the multiple testing burden and thereby found significant associations for additional regulatory variants. We mapped eQTLs in CD4^+^ T cells, CD8^+^ T cells, CD14^+^ monocytes and CD16^+^ neutrophils, accounting for potential confounders including disease status, gender and age (Additional file 4: Supplemental Methods). At a 10 % FDR threshold, we identified 51 genes with a *cis*-eQTL in at least one cell type (Additional file 7). eQTLs have been mapped in CD4^+^ T cells, monocytes and neutrophils in other cohorts [34, 35, 37–40]. Of the genes we identified with eQTLs in these subsets, 56 % were previously reported in at least one of these other cohorts (Additional file 7). eQTL effects on gene expression were found to be generally concordant between IBD patients and healthy controls (Additional file 4: Figure S4). Only six genes, such as the apoptosis inducer *FAS* (also known as CD95; Fig. 3a), had detectable eQTLs across all cell types examined (Additional file 4: Figure S5). Four of these six genes function in the apoptotic pathway by which FAS signals through FADD to activate caspase-induced death and one (*MLKL*) is integral to the necroptotic pathway [67]. TNFSF and TNFRSF genes often appear in clusters throughout the genome due to their origin via gene duplication [68] and therefore many share *cis* elements. Intriguingly, we identified one SNP for which the minor allele was associated with increased neutrophil expression of the TRAIL receptor *TNFRSF10B* (DR5) and decreased monocyte expression of the decoy TRAIL receptor *TNFRSF10C* (DcR1) approximately 34 kbp away (Fig. 3b).

Use of a fixed FDR threshold can lead to underestimation of the extent of eQTL sharing across tissues due to varying power from different sample sizes and effect sizes, as well as random sampling error. A heatmap of the strongest eQTL SNP for each gene in each cell type revealed a greater level of common regulation than evidenced by a rigid FDR threshold (Fig. 3c; Additional file 4: Figure S5). Some genes, such as *TNFSF14* (LIGHT), exhibited strong subset-specific regulation, while others, such as its receptor *TNFRSF14* (HVEM), met our significance threshold in only one subset but showed a trend toward association in other cell types. Clustering these test statistics revealed that the greatest similarity between cell subsets was among T cells, similar to observations at the expression level.

To better understand the complexity of *cis* genetic control over gene expression in TNFSF-related genes, we examined each gene in each cell type with more than one significant *cis*-eQTL SNP. By fitting a linear model with all significant eQTL SNPs for the gene as predictors, we performed exhaustive variable selection to find the most informative set of genetic predictors (Fig. 3d; Additional file 8). Most *cis*-eQTLs could be attributed to a single SNP, while some could be explained by up to four contributing SNPs. Genetic fine-mapping at these loci could clarify whether these multiple contributing SNPs were truly independent or participating in mutual tagging of an un-typed causal variant. Cell types with a greater number of genes with *cis*-eQTLs also exhibited greater tendency to have complex, multi-SNP *cis*-eQTLs.

### eQTLs for TNFSF-related genes are not associated with average gene expression level or enhancer marks

**Fig. 4:**
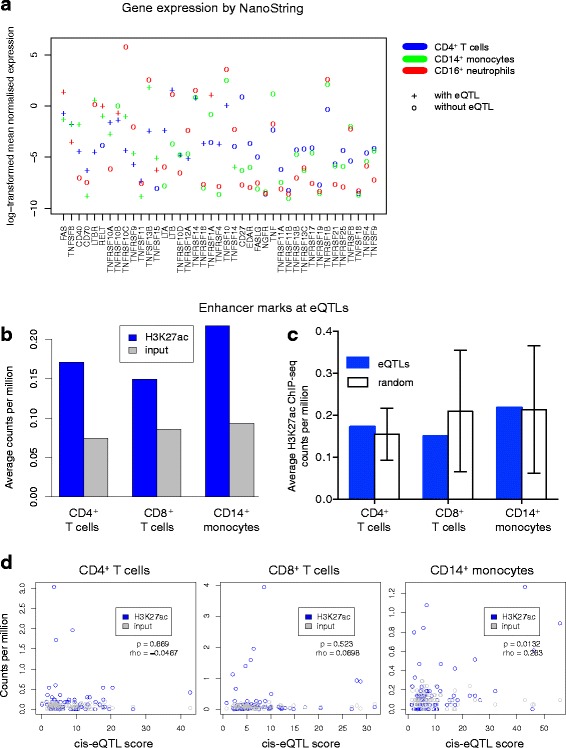
eQTLs are independent of total magnitude of gene expression and not preferentially associated with active enhancer marks. **a** Expression of TNFSF and TNFRSF members was measured by the NanoString nCounter analysis system. Each *point* represents average expression over eight or more individuals, including healthy controls and individuals with IBD. Genes are listed *left* to *right* in order of decreasing number of cell types in which an eQTL was detected. **b** Average H3K27ac ChIP-seq or input DNA sequencing counts per million intersecting TNFSF-related eQTL SNPs in the same cell type are plotted. **c** Average H3K27ac ChIP-seq counts per million intersecting eQTL SNPs in the same cell type are compared with a random distribution of intersections created by selecting the same number of SNPs from the *cis* genomic regions used for eQTL search. *Error bars* represent the standard deviation of 10,000 iterations of random selection. **d** eQTL chi-squared scores from the strongest eQTL SNP for each TNFSF-related gene (regardless of whether the association passed our eQTL significance threshold) are compared with H3K27ac ChIP-seq or input DNA sequencing counts per million at the same SNPs. Spearman correlation coefficient (*rho*) and correlation *p* values (*p*) are indicated for H3K27ac ChIP-seq counts per million versus eQTL score

In microarray measurements, probe effects can hinder comparisons of expression levels between genes and saturation and noise can impact measurements at the extremes [69, 70]. To understand how average gene expression related to eQTL detection, we utilised expression measurements of TNFSF and TNFRSF members acquired by the Nanostring nCounter technology, which provides a count-based measurement without nucleotide amplification steps. These data encompassed three of the four cell types investigated in a similar cohort of healthy controls and IBD patients [58] (Fig. 4a). Discovery of an eQTL for a gene in a given cell type was not related to its average expression in relation to other genes or other cell types. Presence or absence of eQTLs may instead be regulated by other factors such as transcription factor expression or chromatin state.

To investigate the possibility that eQTLs were associated with chromatin marks of enhancers, we used primary leucocyte data from the NIH Roadmap Epigenomics Project. Acetylation of histone 3, lysine 27 (H3K27ac) has been shown to delineate active enhancers [71]. Extracting the most significantly associated eQTL SNP for each gene in each cell type with a *cis*-eQTL (FDR < 0.1), we examined these loci in H3K27ac ChIP-seq data from primary CD4^+^ T cells, CD8^+^ T cells and CD14^+^ monocytes. In all cell types, eQTLs were, on average, enriched by H3K27ac immunoprecipitation compared with input control DNA (Fig. 4b; Additional file 4: Figure S6a). However, randomly sampled SNPs from the same *cis* regions around TNFSF-related genes showed acetylation similar to that of eQTL SNPs (Fig. 4c), suggesting that H3K27ac marks are not specific for eQTLs but rather are characteristic of genic regions. To control for the fact that we did not have fine-mapped eQTLs, we repeated this comparison to include all SNPs tagged (LD r^2^ ≥ 0.8) by our eQTL SNPs (Additional file 4: Figure S6b) and again found no difference in acetylation compared with a random selection of SNPs from the same genic regions. To examine whether eQTL strength correlated with acetylation level, the most significant eQTL SNP for each gene (regardless of whether the eQTL passed our FDR threshold) was extracted. The eQTL chi-squared association statistics were then plotted against acetylation at the same loci (Fig. 4d). In monocytes, but not in other cell types, we found correlation between eQTL strength and H3K27ac enrichment. Indeed, relatively few significant eQTLs were strongly acetylated in the cell type of their discovery, though many monocyte eQTLs did exhibit greater acetylation in monocytes than in other cell types (Additional file 4: Figure S6c, d). Together, these data demonstrate that, within our gene set, eQTLs are not enriched for active enhancer marks over other SNPs within the genic regions.

### TNFSF-related eQTLs are associated with a variety of autoimmune and autoinflammatory diseases

**Fig. 5:**
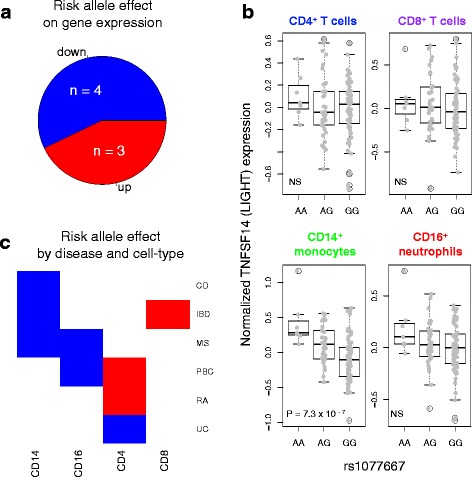
Immune-mediated disease risk alleles can either increase or decrease TNFSF-related gene expression. **a** Autoimmune and autoinflammatory disease GWAS hits tagged by TNFSF-related *cis*-eQTLs were identified and the directions of effect of the risk alleles on expression are plotted. Disease-associated SNPs that are eQTL SNPs in multiple cell types or are associated with multiple diseases are counted only once. “*n*” indicates the number of SNPs in each slice of the pie. **b** TNFSF14 expression is plotted against MS-associated SNP rs1077667 genotype. Allele A is protective. *P* values are provided for eQTLs with FDR < 0.1; *NS* indicates not significant. **c** Disease-associated eQTL SNPs depicted in **a** are plotted by eQTL cell type and disease association. Effect directions are coloured as in **a**. SNPs associated with more than one disease are plotted once per disease

Loci near 24 % of autosomal TNFSF-related genes have been associated with autoimmune and inflammatory diseases by GWAS (Additional file 3, “Mapped Genes” column), resulting in highly significant enrichment of these gene loci with autoimmune diseases (Fisher’s exact test *p* = 1.4 × 10^−10^). However, these SNPs are attributed to genes by physical proximity, not by evidence of functional relationship. To understand the role of SNPs that affect gene expression in immune-mediated disease susceptibility, we searched for TNFSF-related gene eQTLs among autoimmune and autoinflammatory disease-associated SNPs in a comprehensive database of previous GWASs (the NHGRI GWAS Catalog; Additional file 9). We found that approximately equal numbers of disease risk alleles were associated with increased and decreased gene expression (Fig. 5a). For example, the multiple sclerosis (MS) protective allele near the co-stimulatory ligand LIGHT (encoded by *TNFSF14*) was associated with increased expression of this molecule in monocytes (Fig. 5b). Examining the cell types in which these GWAS SNPs were eQTLs revealed a variety of effects across diseases and cell types, potentially suggesting protective effects of TNFSF-related genes in myeloid cells but disease risk effects of members of this signalling network in T cells (Fig. 5c). Such cellular diversity emphasises the distinct influences of TNFSF-related gene variants in autoimmune and autoinflammatory disease onset. A caveat to this analysis is that because many of the GWASs in the GWAS Catalog are not fine-mapped, we cannot confirm whether eQTL and disease association signals at the same locus are due to the same causal variant.

Given the immunological roles and interconnected nature of the TNFSF-related gene network, we wished to examine genetic association of the whole gene set with autoimmune and autoinflammatory disease. Assignment of eQTL SNPs to genes for gene set analysis has previously been proposed to ensure functional relevance of variants used in gene set testing of genetic data [72]. Using this strategy, we re-analysed available GWAS data from eight autoimmune and autoinflammatory diseases for association with TNFSF-related genes. A TNFSF eQTL SNP set was created by combining the strongest significant eQTL SNP for each TNFSF-related gene in each cell type and then filtering these SNPs for relative independence. *LTA*, *TNF* and *LTB* are located within the major histocompatibility complex (MHC) and SNPs near these genes might, therefore, appear disease-associated due to LD with MHC variants that are strongly associated with disease. *TNF* and *LTB* lacked eQTLs and were therefore not included in the TNFSF eQTL SNP set. Disease association with the *LTA* eQTL SNP could not be proven independent of the strong MHC association with many diseases and thus the *LTA* eQTL was excluded from the set. The inflation factor (λ) for disease association with the TNFSF eQTL SNP set was computed and compared with that from genome-wide SNPs thinned for LD (Table 1; Additional file 4: Figure S7). This analysis revealed inflated λ values for TNFSF eQTL SNP set association with six of the eight diseases, in particular CD and Behçet’s disease (BEH), but not type 1 diabetes (T1D) or anti-neutrophil cytoplasmic antibody (ANCA)-associated vasculitis (AAV).

**Table 1:** Autoimmune and autoinflammatory diseases show widespread association with functional variants of the TNFSF network

Disease	λ whole genome^a^	λ eQTL SNP set^b^	eQTL SNP set association *p* value^c^
Behçet’s disease (BEH)	1.05	2.48	0.0002
Crohn’s disease (CD)	1.04	2.39	0.0003
Multiple sclerosis (MS)	1.06	1.59	<0.0001
Primary biliary cirrhosis (PBC)	1.06	1.49	<0.0001
Rheumatoid arthritis (RA)	1.02	1.48	0.003
Ulcerative colitis (UC)	1.04	1.44	0.0021
ANCA-associated vasculitis (AAV)	1.11	1.25	0.17
Type 1 diabetes (T1D)	1.04	1.00	0.77

^a^ λ values were calculated for the whole genome filtered for relative SNP independence

^b^ λ values were calculated for the TNFSF eQTL SNP set defined as follows: for each TNFSF-related gene in each cell subset with a significant *cis*-eQTL (FDR < 0.1), the strongest eQTL SNP was identified; SNPs with LD r^2^ ≥ 0.8 with these eQTL SNPs were then extracted from the GWAS dataset and filtered for relative independence

^c^ Permutation-based *p* values for TNFSF eQTL SNP set association with disease were calculated from the same set of SNPs as in the TNFSF eQTL SNP set^b^

To test total association of TNFSF-related genes with disease, we employed a self-contained method for gene set disease association analysis. Self-contained analyses compare gene set association with a simulated null distribution of no association. This answers the question of whether the gene set is associated with disease, without requiring competitive comparisons with other gene sets that we cannot a priori assume are not associated with disease. To this end, we combined disease association test statistics across the TNFSF eQTL SNP set and used phenotype permutation to simulate a null distribution, modelling our method after that of Sun et al. [64]. Total disease association of a SNP set can be driven by either a single, highly associated variant or multiple, less associated variants. This analysis found significant gene set association with the same six diseases as the inflation factor comparison, particularly with primary biliary cirrhosis (PBC), MS, CD and BEH (column 3 in Table 1; Additional file 4: Figure S8).

**Fig. 6:**
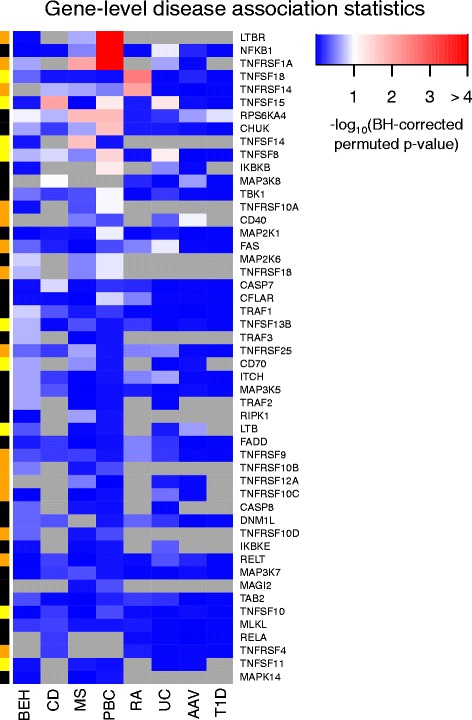
Genes regulated by disease-associated eQTL SNPs differ across diseases. Within each disease, permutation-based *p* values for gene-level disease association were calculated by combining the strongest significant *cis*-eQTL SNPs in each cell type. As in Table 1, proxy eQTL SNPs in each genetic dataset were filtered for relative independence before computation of a permutation-based disease association *p* value. *P* values were corrected within each disease by the Benjamini–Hochberg FDR method. The heatmap represents the negative logarithm of these corrected values such that genes marked in *white*-to-*red* shades show disease association, FDR < 0.1. *Grey* indicates no data available because the GWAS dataset did not include SNPs that tagged eQTLs for this gene with LD r^2^ ≥ 0.8. Gene colours on the *left side* correspond to TNFSF ligands (*yellow*), TNFRSF receptors (*orange*) and adaptors/signalling molecules (*black*) as in Fig. 2. Genes are presented in order of decreasing association with any disease

To reveal whether gene set association results were driven by eQTLs for particular genes or a more general inflation of test statistics for these variants, we examined disease association of eQTL SNPs at the gene level by the same method (Fig. 6). After correcting for the number of genes tested, most TNFSF-related genes were not found to be significantly associated with disease (Fig. 6; blue shades indicate Benjamini–Hochberg FDR > 0.1). Most disease-associated genes were unique to individual diseases but associations with eQTL SNPs for *TNFRSF1A*, *TNFSF15* and *RPS6KA4* were shared across two or three diseases, similar to results observed by Parkes et al. [73] in a comparison of genome-wide significant loci across diseases. A striking cluster of genes was associated with PBC, including *LTBR* (TNFR3), *TNFRSF1A* (TNFR1), *NFKB1* (the p50 subunit of the classical NF-κB transcription factor complex), *CHUK* (NF-κB inhibitor kinase α) and *IKBKB* (NF-κB inhibitor kinase β), that all play roles in TNF and lymphotoxin alpha (LTA) signalling.

## Discussion

We have investigated the contribution of genetic variation in TNFSF-related genes to gene expression and susceptibility to autoimmune and autoinflammatory disease. Previous studies have examined the intersection of a similar variety of genomic data [16] but our approach is unique in its hypothesis-driven investigation of a biological pathway. Targeted eQTL analysis revealed extensive and variable genetic regulation. Shared eQTLs across cell types in the FAS-mediated apoptosis and necroptosis pathways suggest universality of genetic regulation of programmed cell death responses. In the case of TRAIL receptor expression, opposing regulation of competing signalling and decoy receptors by the same eQTL implies enhanced upregulation of this TRAIL-induced death pathway in individuals carrying the minor allele. eQTL detection was not dependent on gene expression levels and eQTL SNPs were not preferentially marked by H3K27ac chromatin modifications. It is possible that, under conditions of acute cellular activation, a different relationship between eQTLs and enhancer loci might emerge.

We studied genes in the TNFSF, their receptors and associated signalling proteins based on the strong involvement of genes in this cytokine superfamily with inflammatory processes and their sharing of downstream signalling pathways. The downstream molecules in TNFRSF signal transduction can play roles in additional pathways, such as pattern recognition receptor signalling [74], and thus association of these genes with autoimmune disease may also reflect involvement of additional pathways not addressed in this study. By using eQTL SNPs as previously suggested by Zhong et al. [72], we ensured that the variants defining our gene set were functionally relevant. Through empirical examination of disease association inflation factors and application of a phenotype-permutation-based test for significance, we were able to demonstrate association of TNFSF-related genes with CD, BEH, PBC, MS, UC and RA, often beyond that of known GWAS loci, but we did not find association with AAV or T1D.

The fact that the TNFSF eQTL SNP set exhibited elevated λ in diseases associated by GWASs with only one or two of these variants (Additional file 8) suggests that additional TNFSF-related eQTLs may have subtle effects on disease susceptibility and lead to the observed cumulative disease association. None of the TNFSF eQTL SNPs have been found to be associated with BEH by GWASs, but their cumulative association is highly significant compared with our permuted null dataset. This suggests that insufficient power may have prevented individual variants from reaching genome-wide significance in the original study that we re-analysed. Such associations might reach genome-wide significance if larger cohorts become available. Association of the TNFSF eQTL SNP set with BEH predicts that expression of TNFSF cytokines, receptors and signalling molecules contributes to the pathogenesis of this disease more than has been previously appreciated. Indeed, rare variants in the NF-κB inhibitor *TNFAIP3* (A20) have recently been associated with a BEH-like familial disease [75] and variants contributing to dysregulation of NF-κB signalling may thus also contribute to the more common form of BEH.

GWASs have identified associations between PBC and genomic loci near *LTBR*/*TNFRSF1A* and *NFKB1* (Additional file 3) [76] but the genetic associations with the NF-κB inhibitor kinase subunits *CHUK* and *IKBKB* that we describe here have not been previously established. Interestingly, a variant near CHUK has been associated with plasma liver enzyme levels [77] and these two kinases have been found to co-ordinately protect liver bile ducts from inflammatory destruction in mice [78]. Thus, our targeted analysis indicates that such associations may reach genome-wide significance with larger PBC GWASs and further implicates the TNF/LTA pathway in PBC pathogenesis. In fact, evidence of the power of a targeted gene set analysis approach to identify sub-threshold associations is already apparent within this study: eQTL SNPs for *TNFSF15* and *NFKB1* did not meet genome-wide significance in the CD [79] and UC [80] GWAS datasets used for re-analysis, respectively, but did contribute to the SNP set association (Fig. 6) and were later found to be genome-wide significant in larger studies (Additional file 9). These data provide support for the use of such a targeted approach to highlight potential disease-associated genes and pathways.

Finally, we found that while the TNFSF-related SNP set was associated with six of the eight diseases examined, the genes contributing to association with these six diseases often differed. This variation suggests that while the pathway is relevant in many immune-mediated syndromes, particular branches are more influential in specific diseases, shedding some light on their independent aetiologies.

## Conclusions

We performed a targeted analysis of TNFSF cytokines, their receptors and signalling molecules to better understand their regulation and association with autoimmune and autoinflammatory diseases. By mapping eQTLs and using these regulatory variants in GWAS gene set analysis, we demonstrated association of TNFSF-related genes with six of the eight immune-mediated diseases examined. Through this hypothesis-driven approach, we have suggested disease association of this gene set beyond individual variants identified in genome-wide SNP association testing.

## Abbreviations

AAV, ANCA-associated vasculitis; ALPS, autoimmune lymphoproliferative syndrome; ANCA, anti-neutrophil cytoplasmic antibody; BEH, Behçet’s disease; CD, Crohn’s disease; ChIP, chromatin immunoprecipitation; CVID, common variable immunodeficiency; EGA, European Genome-phenome Archive; eQTL, expression quantitative trait locus; FDR, false discovery rate; GEO, Gene Expression Omnibus; GWAS, genome-wide association study; H3K27ac, histone 3 lysine 27 acetylation; IBD, inflammatory bowel disease; LD, linkage disequilibrium; LTA, lymphotoxin alpha; MHC, major histocompatibility complex; MS, multiple sclerosis; PBC, primary biliary cirrhosis; RA, rheumatoid arthritis; SLE, systemic lupus erythematosus; SNP, single nucleotide polymorphism; T1D, type 1 diabetes; TNF, tumour necrosis factor; TNFRSF, TNF receptor superfamily; TNFSF, TNF superfamily; TRAPS, TNF receptor associated periodic syndrome; UC, ulcerative colitis

## Additional files

Additional file 1: Autoimmune and autoinflammatory disease terms were curated from the list of disease terms available in the NHGRI GWAS Catalog. (XLSX 51 kb) Additional file 2: Autosomal TNFSF-related genes were identified and probesets extracted from the Hugene1.1St Array. Gene names are from Ensembl release 71. (XLSX 31 kb) Additional file 3: The NHGRI GWAS Catalog was filtered for autoimmune and autoinflammatory disease-associated variants near TNFSF or TNFRSF members or downstream signalling molecules (both “Mapped” and “Reported Gene” categories). (XLSX 16 kb) Additional file 4: This file includes Supplemental Methods and Figures S1–S8 with legends. (PDF 954 kb) Additional file 5: eQTL samples are broken down by cell type and diagnosis. (XLSX 36 kb) Additional file 6: GWAS datasets were used for hypothesis-driven gene set analysis. (XLSX 40 kb) Additional file 7: The best-associated SNP is listed for each *cis*-eQTL (FDR < 0.1). Studies that have previously identified eQTLs for these genes in the same cell types are tabulated. (XLSX 56 kb) Additional file 8: Exhaustive variable selection to minimise the Bayesian information criterion (BIC) was used to determine the best model of SNPs for each *cis*-eQTL (FDR < 0.1). (XLSX 33 kb) Additional file 9: GWAS hits tagged by TNFSF-related gene eQTLs (Additional file 7) were examined for risk allele effects. SNPs associated with gene expression in multiple cell types are repeated, one line per cell type. Duplicate associations from different studies were removed in plotting Fig. 5 but are retained here due to different references, *p* values and odds ratios. (XLSX 55 kb)
