# Supplementary material for: Targeted genomic analysis reveals widespread autoimmune disease association with regulatory variants in the TNF superfamily cytokine signalling network
Source: Genome Med. 2016 Jul 19;8:76. doi: 10.1186/s13073-016-0329-5 (PMC4952362; doi:10.1186/s13073-016-0329-5)
Supplement: Additional file 4: — This file includes Supplemental Methods and Figures S1–S8 with legends. (PDF 954 kb) [file 13073_2016_329_MOESM4_ESM.pdf]

Supplemental Methods and Figures for

Targeted genomic analysis reveals widespread autoimmune disease association with regulatory variants in the TNF superfamily cytokine signalling network

Arianne C. Richard[1,2], James E. Peters[1], James C. Lee[1], Golnaz Vahedi[3], Alejandro A. Schäffer[4], Richard M. Siegel[2,*], Paul A. Lyons[1,*], Kenneth G. C. Smith[1,*,†]

* These authors contributed equally to this work.

† Corresponding author

[1] Department of Medicine and Cambridge Institute for Medical Research, The University of Cambridge, Box 139, Cambridge Biomedical Campus, Hills Road, Cambridge CB2 0XY, UK.

[2] Autoimmunity Branch, National Institute for Arthritis and Musculoskeletal and Skin Diseases, National Institutes of Health, Bethesda, MD 20892, USA.

[3] Department of Genetics, Institute for Immunology, Perelman School of Medicine, University of Pennsylvania, Philadelphia, PA 19104, USA.

[4] Computational Biology Branch, National Center for Biotechnology Information, National Institutes of Health, Bethesda, MD 20894, USA.

Supplemental Methods

NHGRI GWAS Catalog search for TNFSF-related genes and intersection with eQTLs

The NHGRI GWAS Catalog [1] was filtered at $p < 5 \times 10^{-8}$ (genome-wide significance) for variants in or near (as defined by the catalogue) any autosomal member of the TNFSF, TNFRSF or their downstream signalling molecules (Additional file 2) associated with any autoimmune or autoinflammatory disease (search terms derived from [2] are in Additional file 1). To test enrichment of TNFSF-related gene associations in autoimmunity and autoinflammation versus other diseases, genes only in the "Mapped Genes" category of the catalogue were considered to avoid reporting bias of immune-related genes. Results were separated by disease trait and genomic regions were counted only once per disease trait to avoid over-counting associations found in multiple studies. Fisher's exact test was then used to test enrichment of autoimmune and autoinflammatory disease-associated loci for TNFSF-related genes (overlap of 29 from 711 autoimmune and autoinflammatory disease associations and 58 associations near autosomal TNFSF-related genes among a total of 4890 catalogue associations).

To examine overlap with TNFSF-related cis-eQTLs, the SNP most strongly associated with gene expression was extracted for each TNFSF-related cis-eQTL (FDR < 0.1) in each cell type. The NHGRI GWAS Catalog filtered as in the previous paragraph was intersected with these eQTL SNPs (linkage disequilibrium, LD, $r^2 \geq 0.8$). LD and phasing between eQTLs and GWAS SNPs were calculated from the 1000 Genomes Phase 1 EUR population vcf files [3]. VCFtools [4] was used to convert the files to PLINK format. LD (--ld-window-kb 1000 --ld-window- 99999 --ld-window-r2 0.8) and phasing (--ld) were calculated in PLINK [5, 6].

Sorting peripheral blood subsets from individuals

Peripheral blood was separated over a Histopaque 1077 gradient. Neutrophils were isolated from the granulocyte pellet using CD16 Microbeads (Miltenyi Biotec). Peripheral blood mononuclear cells were split in two fractions. From one fraction, in sequential rounds of positive selection, monocytes followed by $CD4^+$ T cells were isolated using CD14 and CD4 Microbeads, respectively (Miltenyi Biotec). From the second fraction, B cells followed by $CD8^+$ T cells were isolated using CD19 and CD8 Microbeads, respectively (Miltenyi Biotec). RNA was extracted using the AllPrep DNA/RNA Mini Kit (Qiagen).

Gene expression microarray data processing

Samples exhibiting sex discordance or global dimness were excluded before gene expression microarray data processing. For cell type comparison in healthy controls, gene expression datasets of four cell subsets from five healthy individuals were run in the same microarray batch. For eQTL analysis with healthy controls and IBD patients, expression datasets for each cell subset were analysed separately as there was confounding of cell type and microarray batch. The microarray dataset contained more individuals than genotypes were available for. To increase normalisation robustness, microarray processing for the eQTL study was performed using all available samples of the same diagnoses and cell types as were used in the eQTL study. Probesets were annotated with the pd.hugene.1.1.st.v1 Bioconductor annotation package [7], and samples were processed using the RMA function of the oligo Bioconductor package [8] in R. Quality control was performed by first correcting for microarray batch with the ComBat function of the sva Bioconductor package [9] using diagnosis (Control, CD, or UC), gender, and age as covariates, and then running the arrayQualityMetrics Bioconductor package [10]. Samples with two or more outlying characteristics were excluded from the datasets.

For the healthy control gene expression analysis, we extracted TNFSF-related gene-level probesets from RMA-preprocessed expression data (Additional file 3). Where there was more than one probeset per gene, we selected the probeset with maximal transcript coverage. Where there was equal transcript coverage, a probeset was chosen at random. Heat maps were generated using the heatmap.2 function of the gplots R package [11].

For eQTL analysis, the PEER R package [12] was used instead of ComBat to remove latent as well as known technical and biological confounders. Expression data was adjusted with PEER, specifying batch, gender, diagnosis, and age as known potential confounders, and accounting for 30 hidden factors. Microarray probesets were then filtered as described in the previous paragraph.

Genotype data processing for eQTL analysis

Within each of the two genotyping batches, samples were removed based on the following criteria: differing from pre-sequencing Sequenom typing (concordance < 0.9), duplication (concordance > 0.98 with another sample), inferred sex ambiguous or conflicting with provided sex, call rate < 0.95, or mean normalised magnitude of intensity < 0.9. All subsequent data processing was performed in PLINK [5, 6] except ethnicity principal component analysis (PCA). Within each batch, SNPs and samples were filtered for genotyping rate above 95%, minor allele frequency above 1%, and Hardy-Weinberg equilibrium p-value greater than 10^{-8}. The two batches were then merged and the same filters applied. Heterozygosity was calculated and samples with inbreeding f-statistics outside three standard deviations from the mean f-statistic were removed. Identity-by-state was calculated, and duplicate samples were removed, keeping the duplicate with the fewest missing genotypes. To verify the homogeneity of sample ethnicities, sample genotypes were compared to hapmap3 genotypes [13, 14]. Hapmap3 founder genotypes were filtered for SNPs common to our dataset on the same strand, combined with our data, and thinned to

5% of the original SNP coverage. Ethnicity PCA was performed in R, using the snpStats Bioconductor package [15]. Ethnicity principal components 1 and 2 were plotted and our dataset visually examined for outliers. One outlier was removed. Only autosomal SNPs were retained for eQTL analysis.

Variable selection for multiple cis SNPs contributing to cis-eQTLs

For each gene with more than one significant cis-eQTL SNP in a given cell type, all SNPs associated with expression of that gene (FDR < 10%) were included as predictor variables in a linear model. In an exhaustive model search, all possible variable subsets were evaluated for Bayesian information criterion (BIC) using the regsubsets function from the leaps R package [16]. The model with minimum BIC was chosen.

Nanostring nCounter measurements and data processing

RNA from $CD4^+$ T cells, $CD14^+$ monocytes, and $CD16^+$ neutrophils from healthy controls and IBD patients was previously measured by the nCounter Analysis System (Nanostring Technologies, Seattle, Washington, USA) [17]. Samples were from the same cohort as those used for eQTL mapping, with 11/14 $CD4^+$ T cell samples, 12/14 $CD14^+$ monocyte samples, and 8/12 $CD16^+$ neutrophil samples shared between the Nanostring and eQTL datasets. Hybridisations were carried out with 100 nanograms of RNA for 17 hours before scanning. The custom nCounter probe set included all TNFSF and TNFRSF member genes (but not downstream signalling molecule genes), among other test and control genes. Samples were first normalised for hybridisation efficiency by the geometric mean of positive control probes. No normalisation factors fell outside of the acceptable range of 0.3-3. Normalised counts from each sample were then further normalised to *CNOT1* expression, which was previously found to be a reasonable control gene in the cell types examined [17].

Processing and analysis of H3K27ac ChIP-seq data

For each cis-eQTL (FDR < 0.1), the strongest cis-eQTL SNP was extracted and hg19 coordinates mapped [18]. H3K27ac ChIP-seq or input DNA sequencing reads overlapping these loci were counted using the bedtools [19] intersect function and normalised per million reads. To examine enrichment of H3K27ac ChIP-seq reads across these cis-eQTLs compared with other SNPs, all SNPs tested in the eQTL analysis were intersected with the ChIP-seq .bed files. A random distribution of mean counts per million was created in each cell type as follows. The same number of TNFSF-related genes as had eQTLs in that cell type were randomly selected, and a SNP from the eQTL SNP genotyping chip from the cis region of each of these genes was randomly chosen to compute a mean H3K27ac counts per million overlap. This process was repeated 10,000 times. Because our eQTL SNPs were not fine-mapped, we also expanded this comparison to include all SNPs in LD $r^2 \geq 0.8$ with the eQTL SNPs or randomly selected SNPs from the cis region. LD-tagged SNPs were identified using the 1000 Genomes Phase 1 EUR population vcf files [3]. For each eQTL or random SNP, acetylation was counted at the maximally acetylated tagged SNP, and these ChIP-seq counts per million were then averaged for the true data or for each randomly selected dataset. To compare eQTL strength versus acetylation enrichment, we considered the strongest eQTL SNP for each gene in each cell type, regardless of significance level. The expression association statistics (1 degree of freedom chi-squared scores) for these SNPs were then compared with H3K27ac counts per million at the same loci by Spearman correlation. For visualisation, H3K27ac ChIP-seq data was converted to bedGraph format using bedtools2 [19] genomecov function, and tracks were viewed in the UCSC genome browser, hg19 genome build [20].

Processing of genetic data from previous GWAS

Genetic data processing was carried out in PLINK [5, 6] and R using the snpStats Bioconductor package [15]. All (if any) SNPs and samples flagged for removal by the original studies were removed. If cases and controls were genotyped separately, and if this information was provided in the available data, the following filtering steps were performed in each batch separately before combination: genotyping rate at the SNP and individual level above 95%, minor allele frequency above 1%, and Hardy-Weinberg equilibrium p-value greater than 10^{-8}. After combination of cases and controls, the same steps were performed in the whole dataset. For SNPs with minor allele frequency less than 5%, SNP genotype missingness was required to be less than 1%. For GWAS datasets where SNPs were labelled with a manufacturer's IDs, we annotated SNPs with rsIDs. Where more than one non-rsID corresponded to a single rsID, the ID with fewer missing calls was selected. SNP rsIDs were updated [21] and obsolete SNPs were removed [22] before conversion to hg19 chromosome and base position annotation [18].

GWAS datasets were examined for outlying individuals. For GWASs with X-chromosome data provided, individuals with discordant sex calls were excluded. Heterozygosity was calculated and samples were removed if they had inbreeding f-statistics greater or less than three standard deviations from the mean of a normal distribution fit to the f-statistics. Identity-by-state was calculated, and duplicate or related samples (identity > 0.8) were removed, keeping the individual with fewer missing genotypes. To verify a homogeneous ethnic population, data was compared to hapmap3 genotypes [13, 14]. Hapmap3 founder genotypes were filtered for non-ambiguous autosomal SNPs common to each GWAS, thinned to independent SNPs (multiple correlation coefficient < 0.1), and combined with the same SNPs from the GWAS data. Ethnicity PCA was performed as described in the Supplemental Methods section "Genotype data processing for eQTL analysis". Normal distributions were fitted to principal components 1 and 2, and individuals falling more than 3

standard deviations from the mean of either were removed. Plots were remade and examined for ethnic uniformity.

References:

1. Hindorff LA, MacArthur JEBI, Morales JEBI, Junkins HA, Hall PN, Klemm AK et al. A Catalog of Published Genome-Wide Association Studies. Available at: www.genome.gov/gwastudies. Accessed 8 March 2015.

2. American Autoimmune Related Diseases Association. List of Diseases. http://www.aarda.org/autoimmune-information/list-of-diseases/. Accessed 12 April 2015.

3. Center for Statistical Genetics, University of Michigan. 1000G Phase I Integrated Release Version 3 Haplotypes. ftp://share.sph.umich.edu/1000genomes/fullProject/2012.03.14/phase1_release_v3.20101123.snps_indels_svs.genotypes.refpanel.EUR.vcf.gz.tgz (2012). Accessed 3 Dec 2012.

4. Danecek P, Auton A, Abecasis G, Albers CA, Banks E, DePristo MA et al. The variant call format and VCFtools. Bioinformatics. 2011;27(15):2156-8.

5. Purcell S. PLINK v 1.07. http://pngu.mgh.harvard.edu/purcell/plink/.

6. Purcell S, Neale B, Todd-Brown K, Thomas L, Ferreira MA, Bender D et al. PLINK: a tool set for whole-genome association and population-based linkage analyses. Am J Hum Genet. 2007;81(3):559-75.

7. Carvalho B. pd.hugene.1.1.st.v1: Platform Design Info for Affymetrix HuGene-1_1-st-v1. R package Version 3.8.1.

8. Carvalho BS, Irizarry RA. A framework for oligonucleotide microarray preprocessing. Bioinformatics. 2010;26(19):2363-7.

9. Leek JT, Johnson WE, Parker HS, Fertig EJ, Jaffe AE, Storey JD. sva: Surrogate Variable Analaysis. R package version 3.10.0.

10. Kauffmann A, Gentleman R, Huber W. arrayQualityMetrics--a bioconductor package for quality assessment of microarray data. Bioinformatics. 2009;25(3):415-6.

11. Warnes GR, Bolker B, Bonebakker L, Gentleman R, Huber W, Liaw A et al. gplots: Various R programming tools for plotting data. R package version 2.14.2. 2014. http://CRAN.R-project.org/package=gplots.

12. Stegle O, Parts L, Durbin R, Winn J. A Bayesian framework to account for complex non-genetic factors in gene expression levels greatly increases power in eQTL studies. PLoS Comput Biol. 2010;6(5):e1000770.

13. The International HapMap Consortium. The International HapMap Project. Nature. 2003;426(6968):789-96.

14. The International HapMap Project. hapmap3. ftp://ftp.ncbi.nlm.nih.gov/hapmap/phase_3/. Accessed 3 April 2013.

15. Clayton D. snpStats: SnpMatrix and XSnpMatrix classes and methods. R package version 1.16.0. 2014.

16. Lumley T. Using Fortran code by Alan Miller. leaps: regression subset selection. R package version 2.9. 2009.

17. Richard AC, Lyons PA, Peters JE, Biasci D, Flint SM, Lee JC et al. Comparison of gene expression microarray data with count-based RNA measurements informs microarray interpretation. BMC Genomics. 2014;15:649.

18. UCSC Genome Bioinformatics. dbSNP build 137. ftp://hgdownload.cse.ucsc.edu/goldenPath/hg19/database/snp137.txt.gz. Accessed 8 Sept 2014.

19. Quinlan AR, Hall IM. BEDTools: a flexible suite of utilities for comparing genomic features. Bioinformatics. 2010;26(6):841-2.

20. Kent WJ, Sugnet CW, Furey TS, Roskin KM, Pringle TH, Zahler AM et al. The human genome browser at UCSC. Genome Res. 2002;12(6):996-1006.

21. NCBI: dbSNP. ftp://ftp.ncbi.nih.gov/snp/database/organism_data/human_9606/RsMergeArch.bcp.gz. Accessed 30 Nov 2012.

22. NCBI: dbSNP. ftp://ftp.ncbi.nih.gov/snp/database/organism_data/human_9606/SNPHistory.bcp.gz. Accessed 3 Dec 2012.

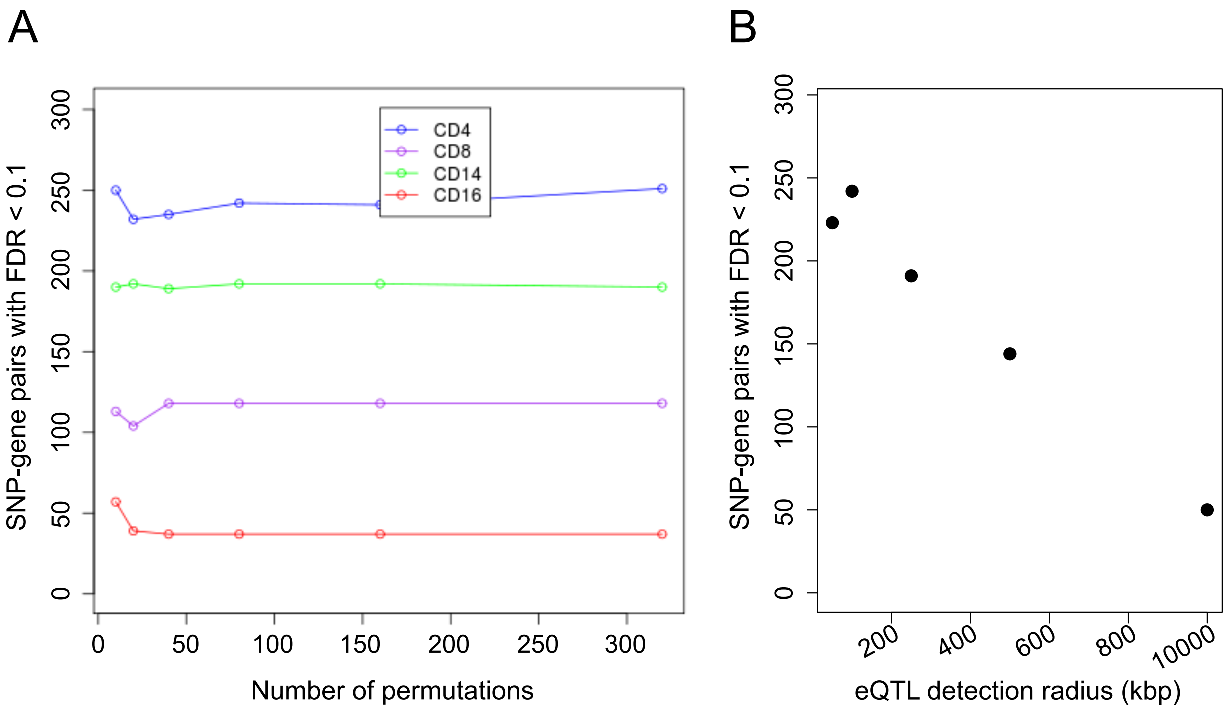

Supplemental Figure 1: **Cis-eQTL parameters. A)** eQTLs were mapped to TNFSF-related genes with a 100 kbp cis radius using different numbers of permutations for FDR calculation. The number of SNP-gene pairs found significant with each number of permutations is plotted for each cell type; CD4=CD4$^+$ T cells, CD8=CD8$^+$ T cells, CD14=CD14$^+$ monocytes, CD16=CD16$^+$ neutrophils. **B**) eQTL analysis for TNFSF-related gene expression in CD4$^+$ T cells was performed using 80 permutations for FDR estimation at varying cis radii. The number of SNP-gene pairs found significant within each cis radius is plotted.

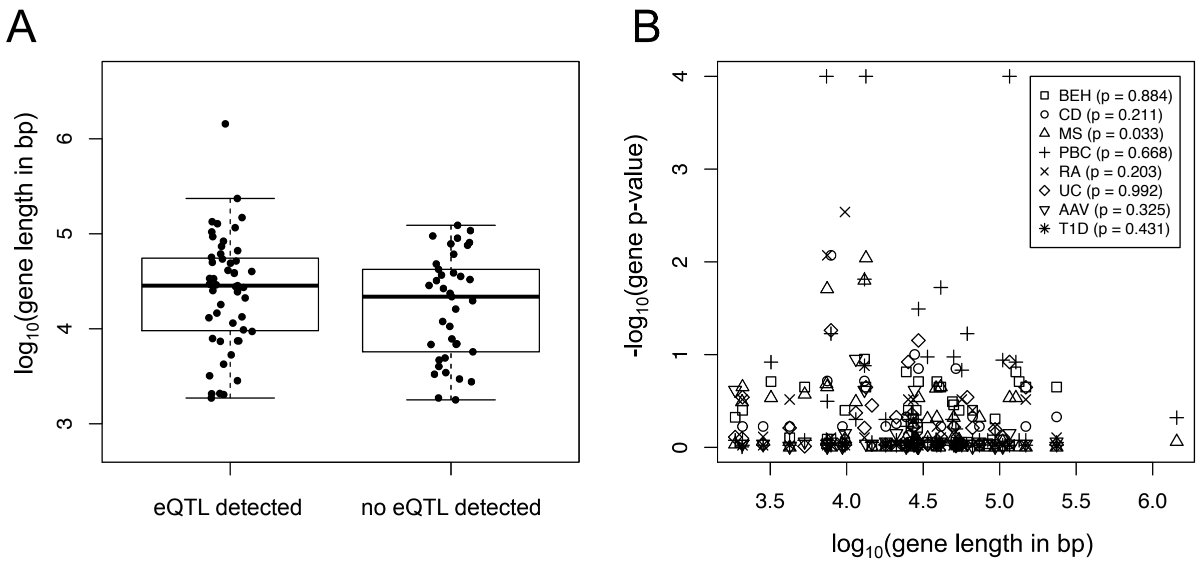

Supplemental Figure 2: **Effect of gene length on disease association statistic.** Because gene-level disease association statistics were dependent on a 2-step process of detecting eQTLs and then computing a permuted association statistic, we tested the impact of gene length on both of these steps. **A**) Boxplot compares the lengths of genes for which an eQTL was detected in any cell type and those for which no eQTL was detected. Distributions are not significantly different by Mann-Whitney test. **B**) Permutation-based p-values for gene association with each disease (values from Figure 6) are plotted against gene length. P-values in upper-right box indicate significance of Spearman correlation.

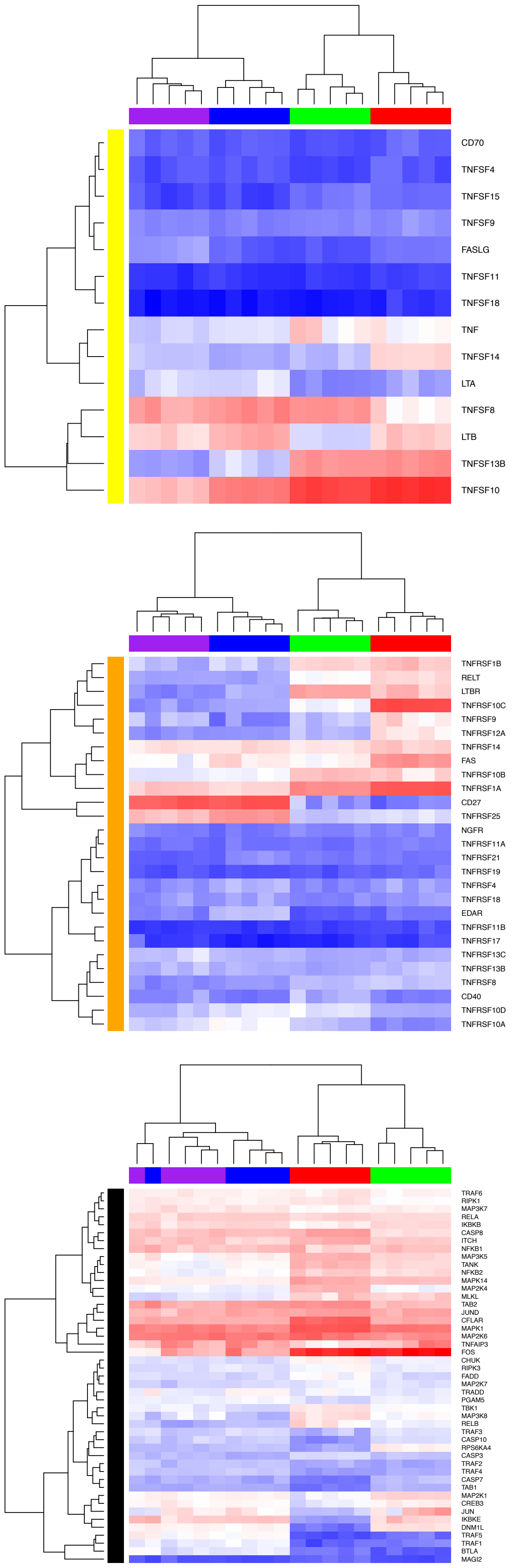

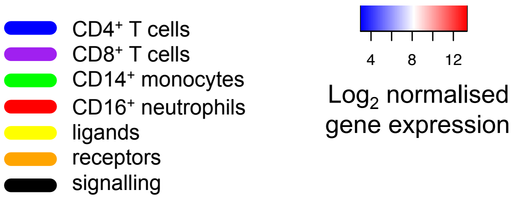

Supplemental Figure 3: **TNFSF-related gene expression.** Expression of TNFSF-related genes in four cell types collected from five individuals is depicted as in Figure 2 for separated TNFSF ligands, TNFRSF receptors, and signalling molecules.

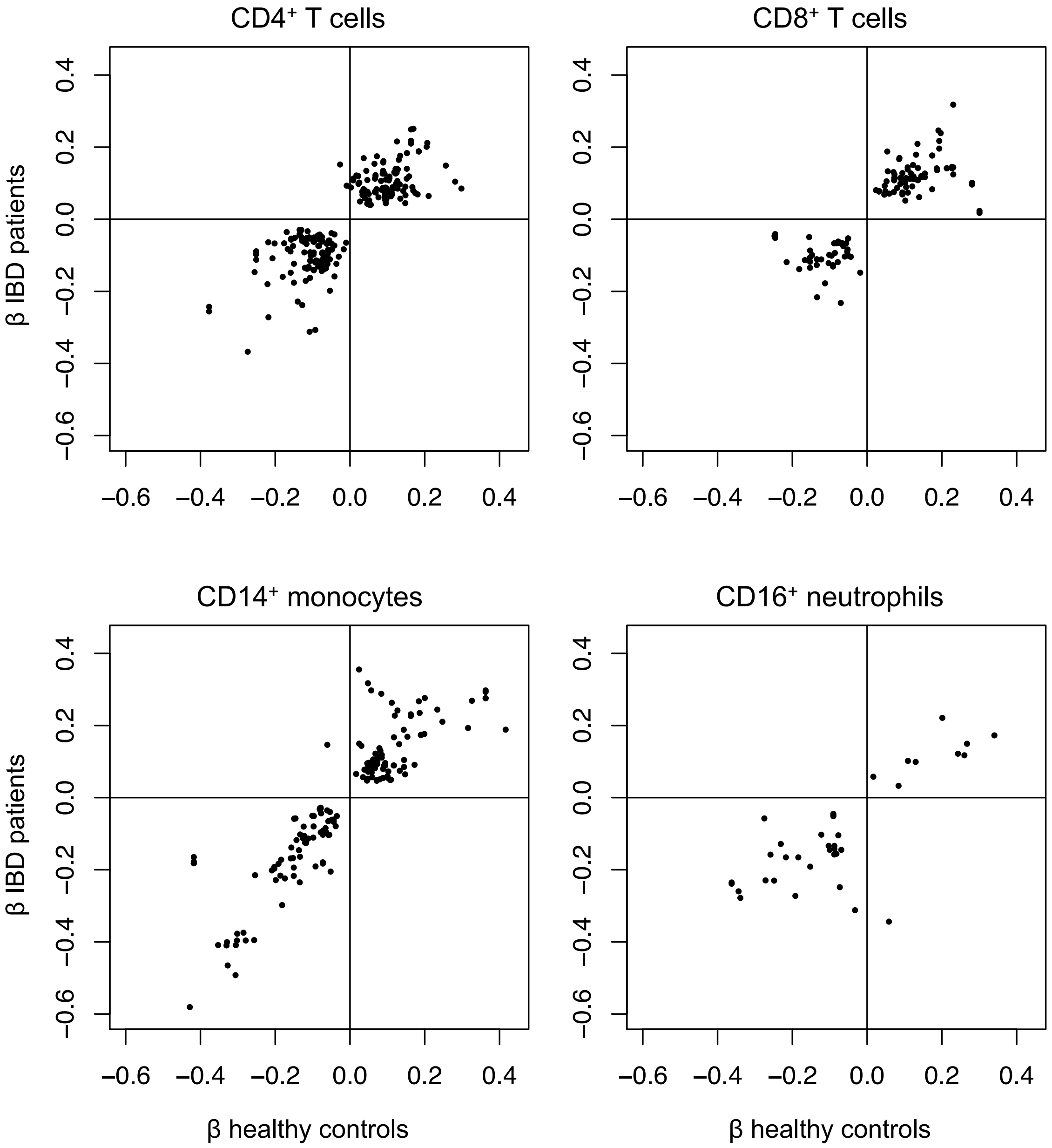

Supplemental Figure 4: **Comparison of eQTL effect size and direction in IBD patients versus healthy controls.** For each eQTL SNP-gene pair (FDR < 0.1) discovered in the combined cohort of healthy controls and IBD patients, we fitted a linear model using just healthy controls or IBD patients. Coefficients for the genotype term (β) are plotted. This value corresponds to the magnitude and direction of effect of genotype on gene expression in the linear model.

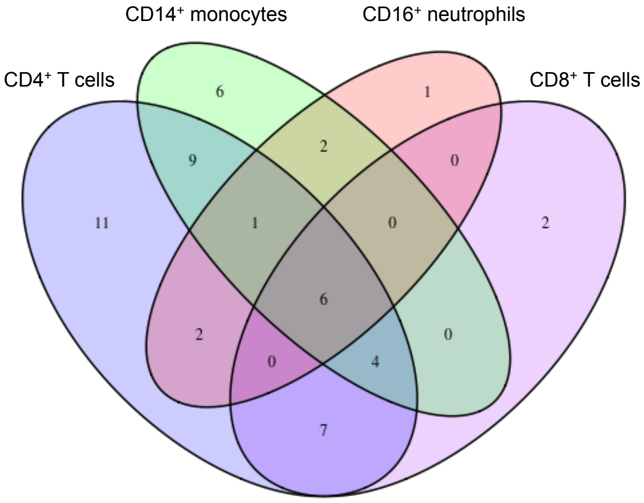

Supplemental Figure 5: **Intersection of cis-eQTL discoveries with strict FDR thresholding.** Venn diagram depicts overlap of genes with a cis-eQTL (FDR < 0.1) between cell types.

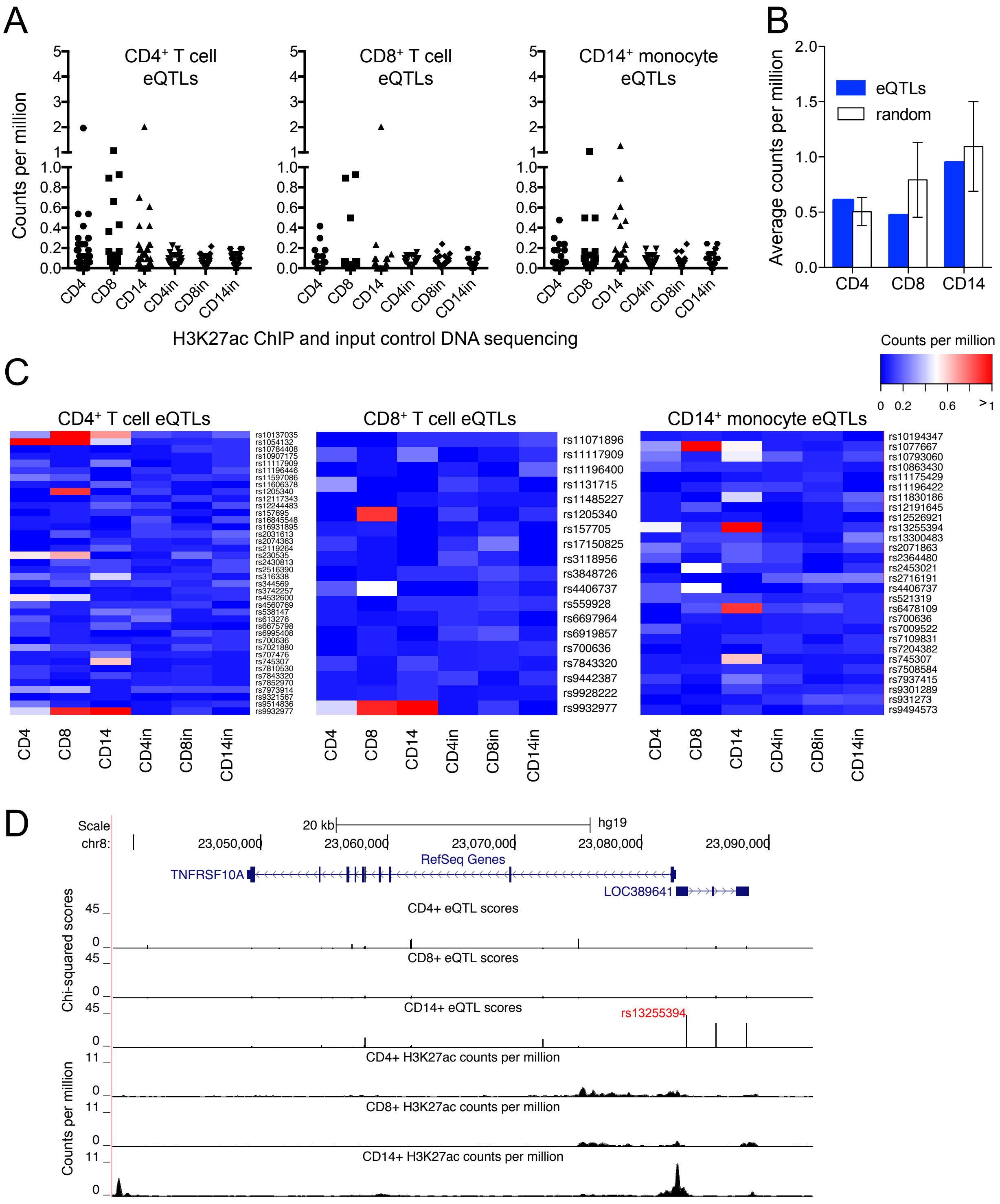

Supplemental Figure 6: **Enhancer marks at cis-eQTLs. A**) Each TNFSF eQTL SNP from CD4⁺ T cells, CD8⁺ T cells, or CD14⁺ monocytes was examined in H3K27ac ChIP-seq and input DNA sequencing data from these same three cell types. “in” indicates input DNA. **B**) As in Figure 4C, acetylation at TNFSF eQTL SNPs was compared with that at randomly selected SNPs cis to TNFSF-related genes. This analysis also considers all SNPs in LD $r^2 > 0.8$ with eQTL or random SNPs. **C**) Heat maps depict the same data as (A) to demonstrate specific SNPs with high H3K27ac ChIP-seq counts per million. **D**) An example of a monocyte cis-eQTL at a monocyte enhancer is shown. TNFRSF10A cis-eQTL chi-squared scores and H3K27ac ChIP-seq counts per million are depicted using the UCSC Genome Browser (http://genome.ucsc.edu). Red indicates the most significant eQTL SNP in CD14⁺ monocytes, which overlaps a monocyte enhancer.

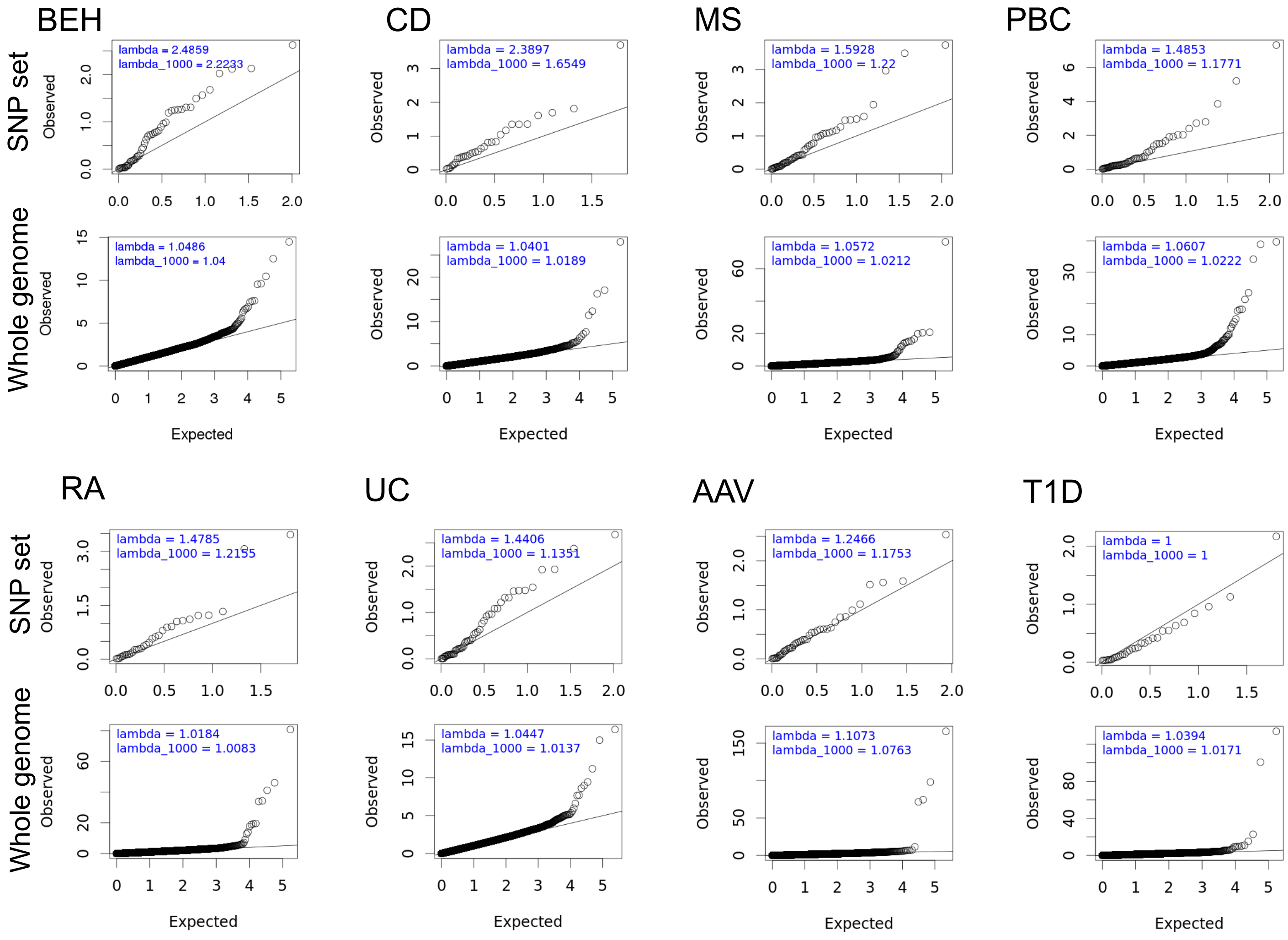

Supplemental Figure 7: **qq-plots.** Qq-plots are the source of values in Table 1. For each disease, SNPs from the whole genome or TNFSF eQTL SNP set were filtered for relative independence, and observed versus expected p-values are plotted. The inflation factor (λ) and the inflation factor normalised for 1000 controls and 1000 cases (λ_{1000}) are indicated.

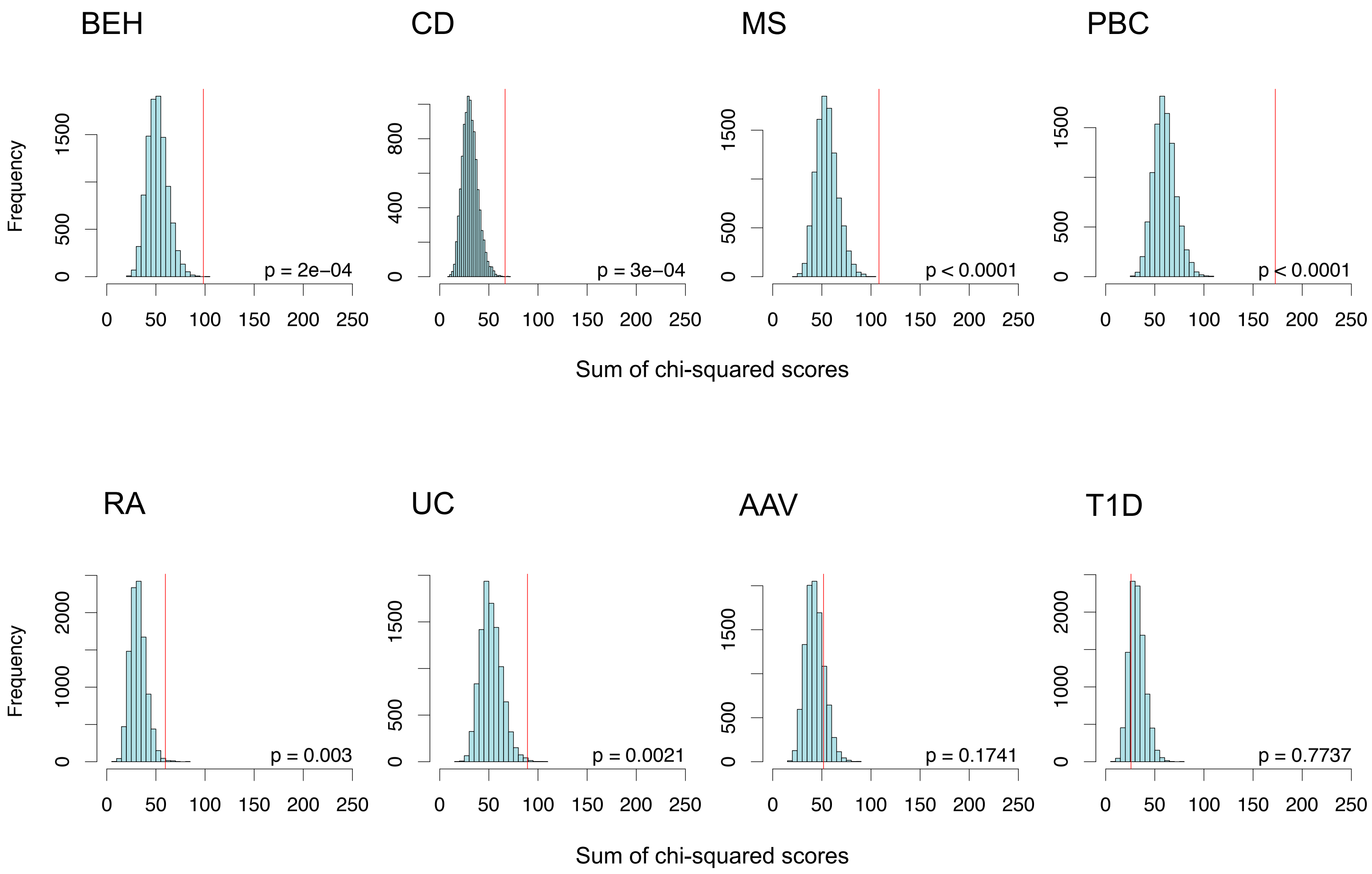

Supplemental Figure 8: **eQTL gene set analysis.** Association statistics for the TNFSF eQTL SNP set compared with a permutation-based null distribution are the source of values in Table 1. For each disease, the blue histogram represents a phenotype-permutation-derived null distribution and the red line represents the statistic in the original data.
